# Unusual Case of Anaplastic Large Cell Lymphoma Presenting as a Breast Mass in a Patient with no History of Breast Implants

**DOI:** 10.1155/2020/7543836

**Published:** 2020-07-23

**Authors:** Yana Ivashkevich, Yaroslav Chernov, Denis Chinenov, Evgeniy Shpot, Alexander A. Bessonov, Arthy Yoga, Kirill A. Lyapichev, Sergej Konoplev

**Affiliations:** ^1^Department of Hematopathology, The University of Texas MD Anderson Cancer Center, Houston, TX, USA; ^2^I.M. Sechenov First Moscow State Medical University, Moscow, Russia; ^3^Breast Cancer Department, National Medical Research Cancer, St. Petersburg, Russia; ^4^Breast Surgical Oncologist, Texas Oncology, Houston, TX, USA

## Abstract

Adenocarcinoma is the most common malignant neoplasm involving breast tissue. In contrast to carcinomas, the other types of malignant neoplasms involving the breast are relatively uncommon. One of the examples of this rare entity is lymphoma. Traditionally, non-Hodgkin lymphomas (NHL) involving the breast are divided into primary lymphoma of the breast and systemic lymphoma, although the distinction could be challenging. Most of NHL involving breast tissue have B cell origin; T cell NHL represents less than 20% of all lymphoma cases. Anaplastic large cell lymphomas (ALCL) involving the breast accounts for even lower percentage of cases. Similar to ALCL involving other sites, there are several main types of ALCL identified: primary cutaneous ALCL and systemic ALCL, which is subdivided into ALK positive and ALK negative subtypes. Relatively recently, an additional distinct subtype of ALK-negative ALCL was described, which is associated with textured breast implants and needs to be considered as a differential diagnosis if patient has a history of breast implants. Here, we report a case of ALCL presented as a breast mass without history of breast implant and discuss similar cases published in the literature.

## 1. Introduction

Adenocarcinoma is the most common malignant neoplasm involving the breast. In the United States of America this year, 276,480 and 2,620 new cases are estimated to be diagnosed in women and men, respectively [1]. In contrast, for nonepithelial neoplasms affecting breast tissue, the other types of malignant neoplasms are exceedingly rare [2]. One of the examples of this rare entity is lymphoma. Traditionally, non-Hodgkin lymphomas (NHL) involving the breast are divided into primary lymphoma of the breast and systemic lymphoma, although the distinction could be sometimes challenging [3, 4]. Most of NHL involving breast tissue have B cell origin; T cell NHL represent less than 20% of all lymphoma cases [3, 4]. Anaplastic large cell lymphomas (ALCL) involving the breast accounts for even lower percentage of cases [3–5]. Similar to ALCL involving other sites, there are several main subtypes of ALCL identified: primary cutaneous, systemic, ALK positive, and ALK negative [6]. Relatively recently, an additional distinct subtype of ALK-negative ALCL was described which is associated with textured breast implants [7]. Here, we report a case of ALCL presented as a breast mass with no history of breast implant and discuss similar cases published in the literature.

## 2. Case Presentation

**Figure 1:**
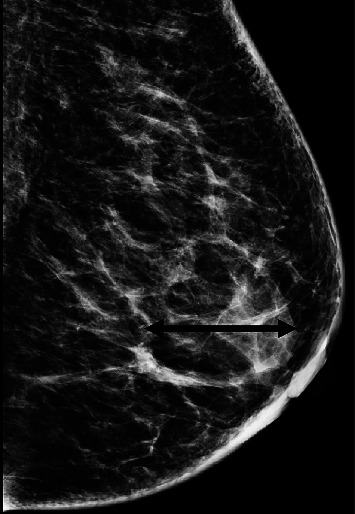
The left breast mammography identifies 2.4 × 2.8 cm mass.

A 45-year-old woman with no prior medical history and no prior breast implants presented to the Emergency Department with breast pain, fever, decreased urine output, decreased appetite and energy level, decreased activity, night sweats, and unintentional weight loss over 25 pounds during the last 6 months. Physical examination revealed temperature 101.7 F, large left breast mass, and a rash on the lateral portion of her left breast. Mammography was performed and showed 2.4 × 2.8 cm mass in the left breast (Figure 1). Chest X-ray revealed bilateral pleural effusions and opacities. PET/CT scan showed extensive hypermetabolic adenopathy in the left axilla with an index region measuring 5 × 5.9 cm and standardized uptake values (SUV) of 17.5. Additionally, the left breast lesions were noted with SUV of 4.9. Superficial cutaneous and subcutaneous uptakes are also identified over the left breast with SUV of 13.8. Hypermetabolic adenopathy was also seen in the right axilla with a conglomerate measuring 2 × 2.5 cm and SUV of 3.7.

**Figure 2:**
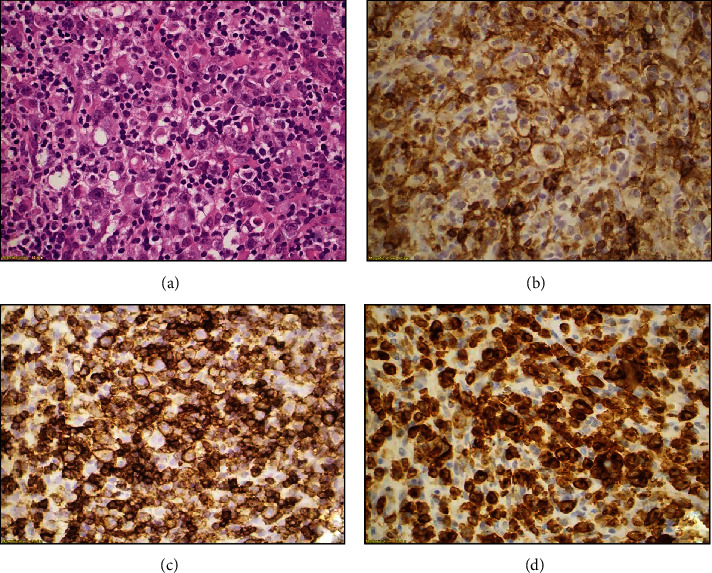
Histologic sections show fragments of soft tissue with extensive infiltration by large, pleomorphic lymphoid cells (a); immunohistochemical studies reveal that neoplastic cells are positive for CD4 (b), CD30 (c), and CD43 (d).

A core needle biopsy of the left breast mass was performed in an outside institution and was subsequently reviewed at our hospital. Histologic sections showed fragments of soft tissue with extensively infiltration by large, pleomorphic lymphoid cells (Figure 2(a)). In some areas, the neoplasm had a starry sky appearance. The neoplastic cells were generally large; a subset of these cells had horseshoe-shaped nuclei consistent with hallmark cells. Mitotic figures and areas of necrosis were identified. Additionally, we reviewed immunohistochemical stains which were provided by the outside institution. The neoplastic cells were found to be positive for CD30 (strong and uniform), MUM1-IRF4 (strong), CD31, cyclin D1 (subset, weak), and Bcl-6 (weak and variable) and negative for ALK, Bcl-2, Pax-5, Sox-11, EBV, CD3, CD5, CD10, CD15, CD20, CD34, CD45/LCA, CD68, CD79a, CD117, CD138, MOC31, pan-keratin, CD5/6, CK903, SOX10, S100, CK8, CKPA, TDT, myeloperoxidase, and kappa and lambda light chains. Ki-67 showed a high proliferation rate of up to 70-80%.

Subsequently, we performed additional immunohistochemical studies using fixed, paraffin-embedded tissue sections in our own laboratory. The neoplastic cells were positive for CD4 (Figure 2(b)), CD30 (Figure 2(c)), CD43 (Figure 2(d)), vimentin, and EMA (focal) and negative for CD21, CD31, CD34, CD35, CD68, CD163, pan-keratin, calponin, desmin, ERG-1, Ber-EP4, and p63. The fluorescence in situ hybridization (FISH) analysis using fixed, paraffin-embedded tissue of this specimen for *IRF4/DUSP22* gene rearrangement was also performed in our institution, and no *IRF4/DUSP22* gene rearrangement was detected.

Based on morphologic features and the results of provided and in-house immunohistochemical studies, we agreed with the diagnosis of ALCL, ALK-negative, established at outside facility.

The staging endoscopy and bone marrow biopsy revealed involvement of antrum and bone marrow, respectively, by ALCL. The patient was started on chemotherapy with brentuximab vedotin, cyclophosphamide, doxorubicin, and prednisone (BV-CHP).

However, immunohistochemical studies performed on bone marrow and stomach biopsies revealed that the neoplastic cells are strongly positive for ALK. Subsequently we repeated ALK immunohistochemical stain on our original, breast biopsy specimen which revealed strong positivity in neoplastic cells. This result was consistent with translocation t(2;5)(p23;q35). We were not able to explain why ALK stain performed at the outside facility gave a false-negative result, but it could be related to technical issues. The diagnosis was revised to ALCL, ALK-positive.

The clinical team was informed, but no changes in patient management were implemented. The patient completed 6 cycles of BV-CHP and demonstrated excellent response with no evidence of disease on the most recent PET/CT and gastroscopy studies 5 months after the initial presentation.

## 3. Discussion

Anaplastic large cell lymphoma with expression of CD30 was originally identified in 1985 by Stein et al. [8]. Subsequently, it was recognized that the most common chromosomal abnormality in ALCL involves anaplastic lymphoma kinase (*ALK*) gene on chromosome 2 [9], which leads to subdivision of this disease to ALK-positive and ALK-negative ALCL. ALCL is a rare disease; it accounts for about 2% of all non-Hodgkin lymphomas [10]. The breast represents a unique location for ALCL because of a distinct subset of neoplasms which are associated with breast implants [7]. ALK-negative ALCL is associated with breast implant neoplasm which was first reported in 1997 by Keech and Creech, although the reconsideration of the truly original published case was recently debated and proposed to be earlier, in 1996 [11, 12]. This type of lymphoma received proper attention only in 2014 when Miranda et al. published a long-term follow-up of 60 patients with ALK-negative ALCL associated with breast implant [7]. Recently, this entity was recognized by WHO Classification [6].

WHO Classification of Tumours of Haematopoietic and Lymphoid Tissues defines ALK-positive ALCL based on anaplastic morphology, a translocation involving the *ALK* gene, and expression of CD30 and ALK protein [6]. ALK-negative ALCL is defined based on similar morphologic and phenotypic features and lack of ALK rearrangement and expression of ALK protein [6]. While the definition includes *ALK* gene rearrangement, practicing pathologists often rely on immunohistochemical studies to distinguish ALK-positive and ALK-negative ALCLs.

However, as the current case illustrates, this approach could lead to a wrong diagnosis if a suboptimal immunohistochemical study gives a false-negative result; therefore, it is prudent to perform additional studies such as FISH to rule out *ALK* gene rearrangement or repeat entity defining immunohistochemical studies.

Although breast implant-associated ALCL is currently receiving increasing attention, it is important to realize that lymphomas involving breast tissue are not limited to the ones associated with breast implants. We reviewed the literature and identified 13 similar cases of systemic ALCL with no prior history of breast implant placement involving breast tissue [3-5, 13-18]. Unfortunately, one of the identified studies did not provide the clinical information about their cases [3]. All available clinical information on similar cases was reviewed and summarized in Table 1.

## 4. Conclusions

ALCL involving the breast is very rare but does occur in both female and male patients.

While cases of ALCL associated with breast implants have been receiving increased attention in the medical community, ALCL also affects patients with no prior history of breast implants and can present as a breast mass. The detection of ALK protein expression by immunohistochemical studies could confirm the diagnosis of ALK-positive ALCL. Although lack of ALK protein expression might be related to technical issues and represent a false-negative result; in this scenario, repeating lymphoma defining markers or FISH studies to rule out *ALK* gene rearrangement might be recommended.

## Figures and Tables

**Table 1:** Systemic ALCL involving the breast in patients with no history of breast implants.

Age (years), sex	Laterality	ALK	Treatment	Outcome	Reference
45, female	Left	Positive	Chemotherapy	Alive 5 mo	Present case
65, male	Right	Positive	Not provided	Alive 18 mo	Gualco et al. [4]
21, female	Right	Positive	Chemotherapy	Death 14 mo	Miranda et al. [5]
35, female	Right	Positive	Chemotherapy, radiation	AWD 18 mo	Miranda et al. [5]
19, female	Right	Positive	Excision, chemotherapy	Alive 23 mo	Sathyanaraya nan et al. [13]
16, female	Right	Positive	Chemotherapy	Death	Daneshbod et al. [14]
33, female	Left	Negative	Chemotherapy+SCT	AWD 30 mo	Kelten et al. [15]
33, female	Right	Positive	Not provided	AWD 30 mo	Krishnan et al. [16]
13, female	Left	Positive	Excision	Death 5 mo	Pereira et al. [17]
92, female	Left	Negative	Excision, chemotherapy	Death 3 mo	Aguilera et al. [18]

AWD: alive with disease.
